# Simultaneous detection of *EGFR* amplification and *EGFRvIII* variant using digital PCR-based method in glioblastoma

**DOI:** 10.1186/s40478-020-00917-6

**Published:** 2020-04-17

**Authors:** Maxime Fontanilles, Florent Marguet, Philippe Ruminy, Carole Basset, Adrien Noel, Ludivine Beaussire, Mathieu Viennot, Pierre-Julien Viailly, Kevin Cassinari, Pascal Chambon, Doriane Richard, Cristina Alexandru, Isabelle Tennevet, Olivier Langlois, Frédéric Di Fiore, Annie Laquerrière, Florian Clatot, Nasrin Sarafan-Vasseur

**Affiliations:** 1 Inserm U1245, Normandie Univ, UNIROUEN, IRON group, Normandy Centre for Genomic and Personalized Medicine, Rouen University Hospital, F-76031 Rouen, France; 2 Department of Medical Oncology, Cancer Centre Henri Becquerel, Rue d’Amiens, F-76038 Rouen, France; 3 Inserm U1245, Normandie Univ, UNIROUEN, Normandy Centre for Genomic and Personalized Medicine, Rouen University Hospital, F-76031 Rouen, France; 4 Department of Pathology, Rouen University Hospital, F-76031 Rouen, France; 5 Department of Genetics, Inserm U1245, Normandie Univ, UNIROUEN, Normandy Centre for Genomic and Personalized Medicine, Rouen University Hospital, F-76031 Rouen, France; 6 Department of Statistics and Clinical Research Unit, Cancer Centre Henri Becquerel, Rouen, F-76038 France; 7 Department of Neurosurgery, Rouen University Hospital, F-76031 Rouen, France; 8 Department of Gastroenterology, Rouen University Hospital, F-76031 Rouen, France

**Keywords:** Glioblastoma, Digital PCR, *EGFR* amplification, *EGFRvIII* variant, Cost-effectiveness

## Abstract

*Epidermal growth factor receptor* (*EGFR*) amplification and *EGFR* variant III (*EGFRvIII*, deletion of exons 2–7) are of clinical interest for glioblastoma. The aim was to develop a digital PCR (dPCR)-based method using locked nucleic acid (LNA)-based hydrolysis probes, allowing the simultaneous detection of the *EGFR* amplification and *EGFRvIII* variant. Sixty-two patients were included. An exploratory cohort (*n* = 19) was used to develop the dPCR assay using three selected amplicons within the *EGFR* gene, targeting intron 1 (EGFR1), junction of exon 3 and intron 3 (EGFR2) and intron 22 (EGFR3). The copy number of *EGFR* was estimated by the relative quantification of EGFR1, EGFR2 and EGFR3 amplicon droplets compared to the droplets of a reference gene. *EGFRvIII* was identified by comparing the copy number of the EGFR2 amplicon to either the EGFR1 or EGFR3 amplicon. dPCR results were compared to fluorescence in situ hybridization (FISH) and next-generation sequencing for amplification; and to RT-PCR-based method for *EGFRvIII*. The dPCR assay was then tested in a validation cohort (*n* = 43). A total of 8/19 *EGFR*-amplified and 5/19 *EGFRvIII-*positive tumors were identified in the exploratory cohort. Compared to FISH, the EGFR3 dPCR assay detected all *EGFR*-amplified tumors (8/8, 100%) and had the highest concordance with the copy number estimation by NGS. The concordance between RT-PCR and dPCR was also 100% for detecting *EGFRvIII* using an absolute difference of 10.8 for the copy number between EGFR2 and EGFR3 probes. In the validation cohort, the sensitivity and specificity of dPCR using EGFR3 probes were 100% for the *EGFR* amplification detection compared to FISH (19/19). *EGFRvIII* was detected by dPCR in 8 *EGFR*-amplified patients and confirmed by RT-PCR. Compared to FISH, the EGFR2/EGFR3 dPCR assay was estimated with a one-half cost value. These results highlight that dPCR allowed the simultaneous detection of *EGFR* amplification and *EGFRvIII* for glioblastoma.

## Introduction

Glioblastoma is the most frequent primary brain tumor in adults, with 125,000 to 150,000 new cases per year worldwide [1]. Despite extensive treatment based on surgery, radiotherapy and chemotherapy combination, recurrence remains the rule with a median overall survival of less than 18–24 months [2]. Diagnosis is commonly based on histopathological examination and characterization of *isocitrate dehydrogenase* (*IDH*)*1/2* mutations [3]. Recent advances also highlighted a key role of other molecular alterations, such as those located on the *epidermal growth factor receptor* (*EGFR*) gene, which is altered in approximately 57% of cases [4]. *EGFR* amplification and *EGFR* variant III (*EGFRvIII*), which is characterized by the deletion of exons 2–7, are the two most frequent *EGFR* alterations in glioblastoma observed in 40–50% and 10% of patients, respectively [4–7]. Interestingly, it has been reported that the presence of *EGFRvIII* is associated with *EGFR* gene amplification in most cases [8]. In this context, specific treatments that directly target the *EGFR* pathway or activate the immune response against *EGFRvIII* have been recently developed using either as a single therapy or in combination with standard treatment [9–12]. Antibody-drug conjugates targeting *EGFR* may improve survival at the time of recurrence in *EGFR*-amplified glioblastoma [13]. In addition, identification of *EGFR* amplification associated with either a *telomerase reverse transcriptase* promoter (*TERTp*) mutation or chromosomal alterations (chromosome 7 gain and chromosome 10 loss) in diffuse or anaplastic astrocytoma has led to a reclassification proposal of grade II-III 1p19q non-codeleted gliomas into glioblastoma-like tumors [14, 15]. Taken together, these data support that the detection of *EGFR* alterations may be considered relevant in patients treated for glioblastoma.

Until now, *EGFR* alterations have been detected by separate methods. Indeed, fluorescence in situ hybridization (FISH) is the gold standard for the detection of *EGFR* amplification, and the use of other methods, such as genomic hybridization (array CGH) or next-generation sequencing (NGS), has also been reported. On the other hand, the detection of the *EGFRvIII* variant, leading to an abnormal expression of ARNm, is performed commonly using RT-PCR-based methods [9].

The development of a specific molecular method allowing the simultaneous detection of *EGFR* alterations may be of interest in glioblastoma. Targeted copy number variation (CNV) detection by digital PCR (dPCR) using locked nucleic acid (LNA)-based hydrolysis probes has recently been shown to be efficient in genetic diseases [16]. LNA-hydrolysis probes are very short nucleotides, and repeated sequences across the human genome may be incorporated in a dPCR amplicon. Gene copy number estimation is then based on the ratio of detected LNA probes between a gene of interest and a reference gene.

In this context, we aimed to develop a novel dPCR assay using LNA-hydrolysis probes located within and outside the region spanning from exon 2 to exon 7 to allow the simultaneous detection of *EGFR* amplification and *EGFRvIII* variant. First, we used an exploratory cohort of patients with glioblastoma to develop a dPCR assay in comparison to FISH for *EGFR* amplification and to an RT-PCR-based method for *EGFRvIII*. In the second step, we tested the ability of our dPCR assay to simultaneously detect these two *EGFR* alterations in an independent validation cohort of patients with glioblastoma.

## Patients and methods

### Patients and tumor samples

The present study is ancillary to the ongoing prospective GLIOPLAK trial (registered in ClinicalTrials.gov, NCT02617745), which is investigating predictive markers of chemo-induced toxicities. A total of 62 patients were recruited from November 2015 to November 2017. Eligible patients were at least 18 years old and had a newly diagnosed and histologically confirmed supratentorial glioblastoma, according to the 2016 WHO classification [3]. Patients received concomitant radiotherapy with temozolomide followed by sequential temozolomide treatment [2]. Tumor samples were obtained during surgery (biopsy, gross-total or partial resection) and processed for routine histopathology, immunohistochemistry and molecular biology experiments. Tumor DNA was extracted from formalin-fixed paraffin-embedded FFPE samples using the Maxwell 16 FFPE Plus LEV DNA Purification® Kit on a Maxwell 16 Instrument® (Promega®, Fitchburg, Wisconsin, United States). *IDH1/2* mutations within exon 4 were analyzed using the ABI PRIM SNaPshot® Multiplex Kit (ThermoFisher Scientific®, Waltham, Massachusetts, USA); *MGMT* promoter (*MGMTp*) methylation was analyzed with the pyrosequencing method (*therascreen* MGMT Pyro®, Qiagen®, ThermoFisher Scientific®).

For the purpose of the present study, the population was divided into two groups: an exploratory cohort, which included the first 19 patients, and a validation cohort, which was based on the next 43 consecutive patients. The exploratory cohort was used to develop the dPCR assay by selecting amplicons and allowing the simultaneous detection of *EGFR* amplification and *EGFRvIII*, according to the standard methods of FISH or NGS and RT-PCR-based methods, respectively. In the second step, we used an independent validation cohort to evaluate the ability of the dPCR assay to detect both *EGFR* alterations.

### Development of dPCR assay

**Fig. 1:**
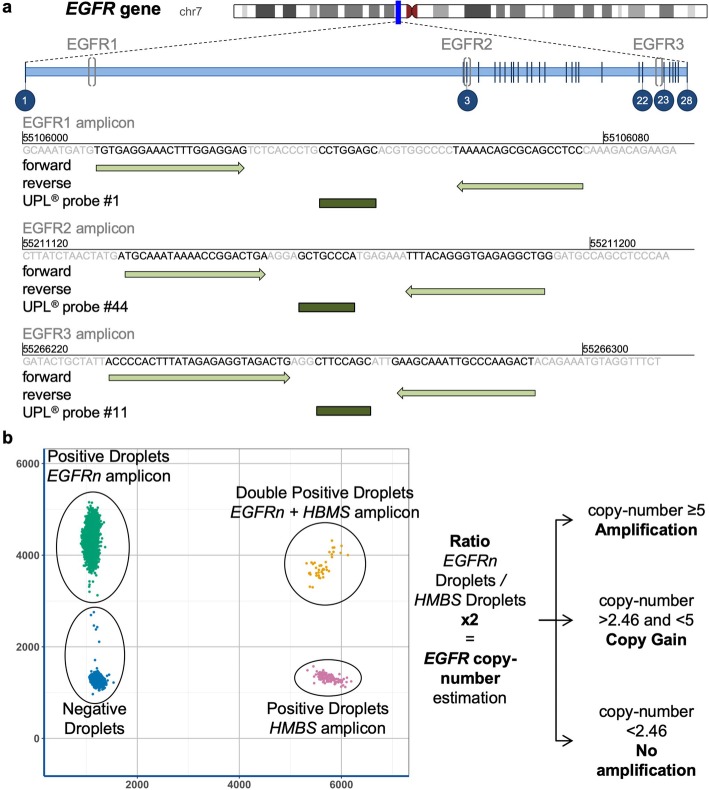
Design of the dPCR assay using LNA-hydrolysis probes for detecting the *EGFR* amplification and *EGFRvIII* variant. **a** Three amplicons were designed within the *EGFR* gene from Universal Probe Library® (Sigma-Aldrich). EGFR1, EGFR2 and EGFR3 are located within three different regions in the gene. EGFR2 is inserted into the deleted region of the *EGFRvIII* variant (deletion of exons 2–7). **b** Two-dimensional cluster plot representing the 6-carboxyfluorescein (FAM)-labeled LNA-based hydrolysis probe for the *EGFR*-targeted sequence (EGFR1, EGFR2 or EGFR3) against the trichloro-phenylcarboxyfluorescein oligonucleotide (VIC)-labeled hydrolysis probe for the *HMBS* amplicon. Droplets are grouped as clusters: FAM/VIC-negative (double-negative droplets, blue), FAM-positive/VIC-negative (green), FAM-negative/VIC-positive (pink), and FAM/VIC-positive (double-positive droplets, orange). The *EGFR* copy number was determined by calculating the ratio of *EGFR* FAM-labeled droplets over the *HMBS* VIC-labeled droplets multiplied by the number of *HMBS* copies (× 2 in the human genome)

According to recently published methods of dPCR using universal LNA-hydrolysis probes from the 96 Universal Probe Library® (UPL, Sigma-Aldrich®, St. Louis, Missouri, USA), three dPCR assays were performed for each tumor sample [16]. These assays used a duplex PCR: one PCR amplicon within the *EGFR* gene and one reference PCR amplicon located in the *hydroxymethylbilane synthase* (*HMBS*) gene*,* a housekeeping gene located in 11q23. Three different amplicons of the *EGFR* gene were designed: the *EGFR1* amplicon located within intron 1 with UPL® probe #1 (reference: 04684974001), the *EGFR2* amplicon located between exon 3 and intron 3 with UPL® probe #44 (reference: 04688040001) and the *EGFR3* amplicon located within intron 22 with UPL® probe #11 (reference: 04685105001) (Fig. 1a). The reference *HMBS* amplicon is located in intron 1 using the forward primer (5′-GGGACAGTGTACCCAAGGTC-3′), the reverse primer (5′-CTGAGGTAAACGGATCTGACG-3′) and a custom trichloro-phenylcarboxyfluorescein oligonucleotide (VIC)-labeled probe (5′-CCAAGAGGCTGAGCAGGACT-3′, ThermoFisher Scientific®). dPCR experiments were performed using a Qx200® droplet digital PCR (ddPCR) System (Biorad®, Hercules, California, USA). ddPCR was run in a final volume of 22 μL with tumor DNA, 10 μl ddPCR Supermix for probes (no dUTP), primers for the *EGFR*- and *HMBS*-targeted amplicons (0.9 μM), 6-carboxyfluorescein (FAM)-labeled LNA-based hydrolysis probe for the *EGFR*-targeted sequence (0.18 μM), and VIC-labeled probe for the *HMBS* amplicon (0.18 μM). Thermal cycling was performed, according to the manufacturer’s instructions: 10 min at 95 °C; then 40 cycles at 94 °C for 30 s and 56 °C for 1 min; and a final step of 10 min at 98 °C. The software QuantaSoft® was used for the interpretation of the profiles. The *EGFR* copy number was determined by calculating the ratio of *EGFR* FAM-labeled droplets over the *HMBS* VIC-labeled droplets multiplied by the number of *HMBS* copies (× 2 in the human genome) (Fig. 1b). CNV assessment was then based on comparisons to the *EGFR* copy number estimation from FFPE control tissues (normal copy number, 1.66–2.46). According to molecular analyses from INTELLANCE trials, glioblastoma was considered to be *EGFR*-amplified if the copy number was greater than or equal to 5 [7]. For *EGFRvIII* identification, we hypothesized that the copy number estimated by the EGFR2 amplicon would be lower than the copy number estimated by EGFR1/EGFR3 for the same sample. The optimal amount of DNA for one dPCR experiment was set at 30 ng.

### *EGFR* amplification detection by fluorescence in situ hybridization

FISH was performed on FFPE tumor samples. After the selection of an area containing more than 70% tumor cells, 4-μm sections were deparaffinized, dehydrated with ethanol and pretreated with Vysis Paraffin Pretreatment IV (Abbott®, Illinois, USA). Hybridization was performed using the EGFR/CEP7 FISH Probe Kit® (Abbott®), according to the manufacturer’s instructions. The probes used covers 303 kb located in the 7p11 region, which contains the *EGFR* gene. The reference probe was located on the centromere of chromosome 7. The post-hybridization step was performed with a Wash Buffer Kit (Abbott®). Samples were considered *EGFR*-amplified when the quantity of fluorescence of the *EGFR*-targeting probe (red fluorescence) was greater than twofold per nucleus than the number of centromere-targeting probes (green fluorescence) and only when at least 15% of tumor cell nuclei were *EGFR*-amplified [7].

### NGS experiments and *EGFRvIII* detection

An Ion Torrent Personal Genome Machine (PGM, Life Technologies®, Carlsbad, California, United States of America) was used for *EGFR* somatic point mutations and *EGFR* amplification detection. Tumor DNA was sequenced using a custom *EGFR*-targeted panel dedicated to highly recurrent altered region of the gene (Additional File: Table S1) [4]. Amplified libraries (Ion AmpliSeq® Library Kit 2.0) were submitted to emulsion PCR using the Ion OneTouch® 200 Template Kit (Life Technologies®) with the Ion OneTouch® System (Life Technologies®). Data analysis was performed using Torrent Suite version 5.4 software (ThermoFisher Scientific®). Reads were mapped to the human hg19 reference genome. Copy-number was estimated using the ONCOCNV algorithm compared to control DNA from healthy subjects [17].

*EGFRvIII* identification was performed using ligation-dependent reverse transcription polymerase chain reaction (LD-RT-PCR), which allows the detection of fusion transcripts and exon skipping [18–20]. RNA was extracted from FFPE tumor samples using the Maxwell® 16 LEV RNA FFPE Purification Kit (reference AS1260, Promega®) and following manufacturer instructions. RNA was converted into complementary DNA (cDNA) using reverse transcription probes located on the end of *EGFR* exon 1 and the start of *EGFR* exon 8; the cDNA was then hybridized. In the case of *EGFRvIII* (deletion of exons 2–7), by adding DNA ligase, a covalent link between the two probes was formed, allowing PCR amplification and subsequent identification by NGS on MiSeq® (Illumina®, San Diego, California, USA).

### Cost-effectiveness estimation

An exploratory cost-effectiveness study was conducted to compare the cost of *EGFR* amplification detection with dPCR with the reference method (FISH). Total costs per patient included reagent costs and medical/technician times.

### Standard protocol approvals, registrations and patient consent

Informed written consent to participate in the study was obtained from all patients. The French National Committee for the Protection of Persons approved the study (RCB ID 2015-A00377–42).

### Statistical analyses

In the exploratory cohort, to assess the equivalence of the *EGFR* copy number estimation between NGS and dPCR, a correlation matrix plot was performed. The sensitivity, specificity and positive and negative predictive values were calculated to assess the diagnostic performance. The gold standard was the FISH results. The copy number difference and its threshold between the EGFR2 assay and the two other techniques to predict the *EGFRvIII* variant were determined using receiver operating characteristic (ROC) curves. In the validation cohort, the sensitivity and specificity of the dPCR assay for detecting *EGFR* amplification was compared to those of FISH. All dPCR and FISH analyses were carried out in a double-blind manner. Statistical analyses and figures were performed using R software (R version 3.5.1, 2018, Vienna, Austria) [21].

## Results

### Baseline characteristics

The characteristics of all patients are detailed in Table 1. Among them, 59 (95%) had wild-type *IDH* (*IDH*-wt) glioblastoma, and 3 had *IDH*-mutated glioblastoma. Overall, *EGFR* amplification was identified in 27 tumors (44%) using FISH; a total of 8 (42%) and 19 (44%) were in the exploratory and validation cohorts, respectively. In the group of patients with an *IDH-*wt glioblastoma, three had a rare histological subtype (1 with giant cell glioblastoma and 2 with gliosarcoma), and none of the three tumors had *EGFR* amplification.

**Table 1:** Clinical and Tumor characteristics

Characteristics		Entire cohort *N* = 62	Exploratory cohort *N* = 19	Validation cohort *N* = 43
Age (years), mean [min. – max.]		56.9 [21–76]	55.5 [28–76]	57.7 [21–72]
Sex	Female	28 (45%)	7 (37%)	21 (49%)
	Male	34 (55%)	12 (63%)	22 (51%)
Surgery	Biopsy	25 (40%)	8 (42%)	17 (40%)
	Resection	37 (60%)	11 (58%)	26 (60%)
Glioblastoma *IDH* wild type		59 (95%)	18 (95%)	41 (95%)
	Giant cell glioblastoma	1 (2%)	1 (5%)	0
	Gliosarcoma	2 (3%)	0	2 (5%)
Glioblastoma *IDH* mutant		3 (5%)	1 (5%)	2 (5%)
*EGFR* amplification by FISH		27 (43%)	8 (42%)	19 (44%)
*MGMTp* methylation	Non-methylated	37 (60%)	13 (68%)	24 (56%)
	Methylated	17 (27%)	6 (32%)	11 (26%)
	Unknown	8 (13%)	0	8 (18%)
*TERTp* mutation	C228T	41 (66%)	14 (74%)	27 (63%)
	C250T	14 (23%)	4 (21%)	10 (23%)
	Wild-type	7 (11%)	1 (5%)	6 (14%)

### Development of a dPCR assay for detecting *EGFR* alterations in the exploratory cohort

**Fig. 2:**
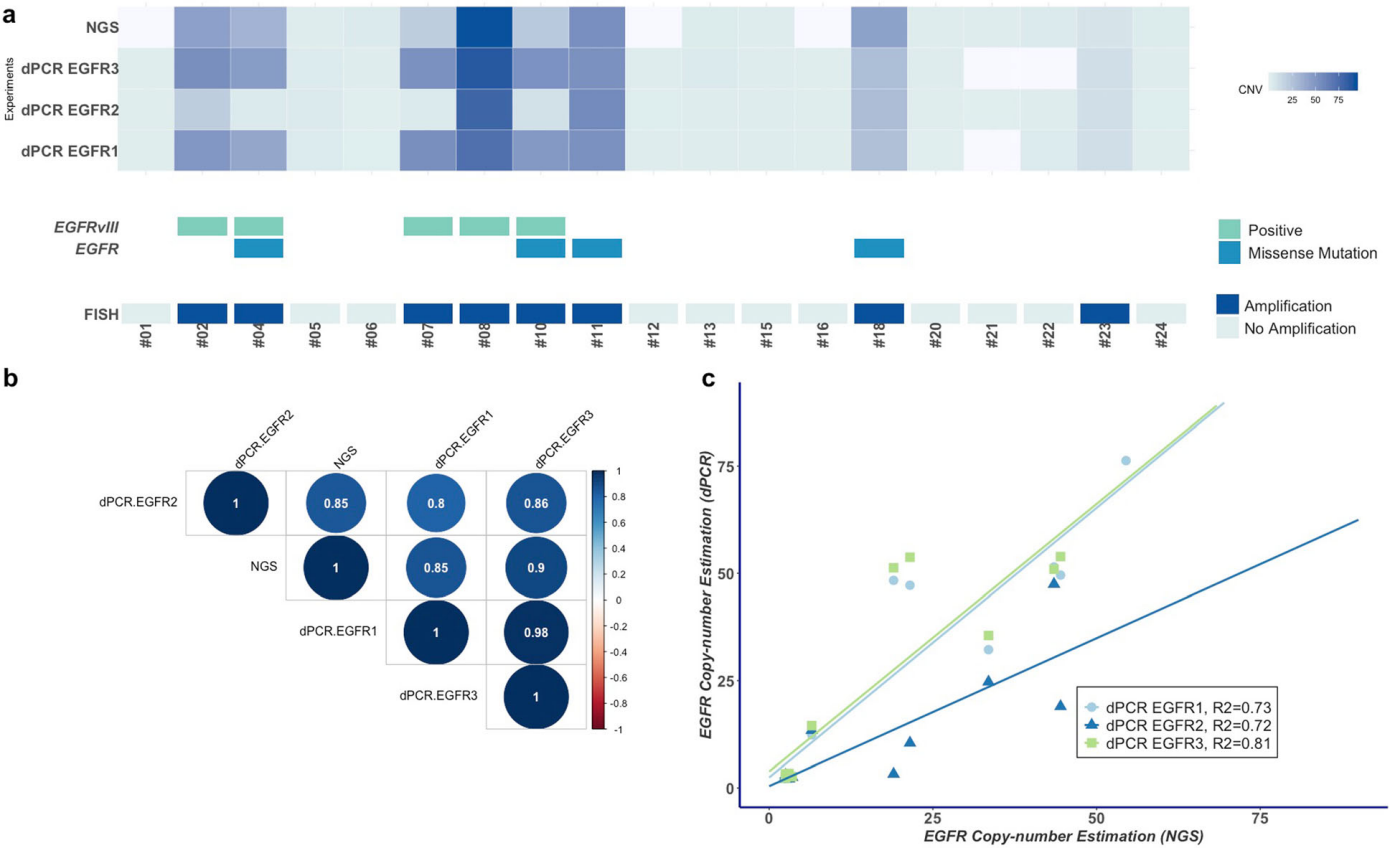
Concordance between the results of the dPCR assay, FISH, next-generation sequencing (NGS) and LD-RT-PCR for the detection of the *EGFR* amplification and *EGFRvIII* variant in the exploratory cohort (*n* = 19). **a** Heatmap of *EGFR* copy number estimated by NGS and the three dPCR assays. Each column represents a tumor sample (n = 19). The blue gradient represents the estimated value of the *EGFR* copy number. There is a strong agreement between the EGFR1 and EGFR2 dPCR assays and NGS. The absence of results using the NGS experiment is indicated by the light gray color. Below the heatmap, the presence of *EGFR* mutations and *EGFRvIII* variant as well as the results of FISH are presented. The presence of somatic mutations was detected by the *EGFR*-targeted NGS panel, and the presence of the *EGFRvIII* variant was detected by LD-RT-PCR. Patient #08 harbors both the *EGFR* amplification and *EGFRvIII* variant with tumor heterogeneity regarding the copy number estimation by dPCR (EGFR1 63, EGFR2 70 and EGFR3 91). **b** Correlation matrix plot of *EGFR* copy number estimation using three dPCR assays (EGFR1, EGFR2 and EGFR3) and NGS (*n* = 16). The dPCR EGFR3 assay results have the highest correlation with the NGS results. On the other hand, the dPCR EGFR2 assay results have the poorest correlation, mainly due to its underestimation of the *EGFR* CNV in the case of *EGFRvIII*-positive glioblastoma. **c** Linear regression curves representing *EGFR* copy number values estimated by NGS (x-axis) and the copy number estimated by the three dPCR assays (y-axis). As expected with the results of the matrix correlation plot, the estimation using the dPCR EGFR3 assay was confirmed to have the best correlation to the NGS estimation

Among the 19 patients in the exploratory cohort, *EGFR* amplification was identified in eight (8/19, 42%) patients using FISH. EGFR1 and EGFR3 assays strictly correlated with FISH results, making it possible to distinguish all *EGFR*-amplified (8/8) and *EGFR*-non-amplified glioblastoma (11/11), with sensitivity, specificity, positive and negative predictive values of 100%. Overall, the mean copy number estimation by dPCR was 25 (range 2–76) using EGFR1 and 29.4 (range 2–98) using EGFR3. Using a threshold of copy number amplification greater than or equal to 5, the mean EGFR1 copy number amplification was 47.5 (range 12.3–76.3), and the mean EGFR3 copy number amplification was 56.1 (14.5–98.3) (Fig. 2a). Using FISH, seven out of the 8 *EGFR*-amplified glioblastomas contained at least 90% cell nuclei with *EGFR* amplification. Interestingly, the remaining *EGFR*-amplified glioblastoma patient (patient #23) had 30% amplified cell nuclei and concordant copy number estimation by dPCR of 12.3 for EGFR1 and 14.5 for EGFR3. The diagnostic performance of EGFR2 was lower with one discordant case; the *EGFR* was not amplified by dPCR but was amplified using FISH (sensitivity of 87.5% and specificity of 100%).

NGS experiments were performed in 16 out of the 19 tumors, and three patients had a tumor DNA quantity that was too low to be analyzed. The 8/16 patients with an *EGFR* amplification detected by NGS were the same as those identified with dPCR assay or FISH. Notably, the correlation coefficient between the copy number calculated by dPCR assays (EGFR1, EGFR2 and EGFR3) and by NGS was higher than 0.8 (Fig. 2b). The copy number estimated by the EGFR3 assay had the highest correlation coefficient with NGS values (correlation coefficient of 0.9 and R-squared of 0.81) (Fig. 2c). The copy number estimation by the EGFR2 assay had the lowest correlation with the EGFR1/EGFR3 dPCR assays and with NGS. Moreover, the mean copy number by the EGFR2 assay was significantly lower than that estimated by the EGFR3 assay (18.8 vs 29.4, *P* = 0.023). Five *EGFR*-amplified glioblastomas had a lower estimated copy number by EGFR2 than those by EGFR1/EGFR3 assays; in one patient (patient #07), an EGFR2 copy number was estimated as 3.3, which was below the established cutoff.

**Fig. 3:**
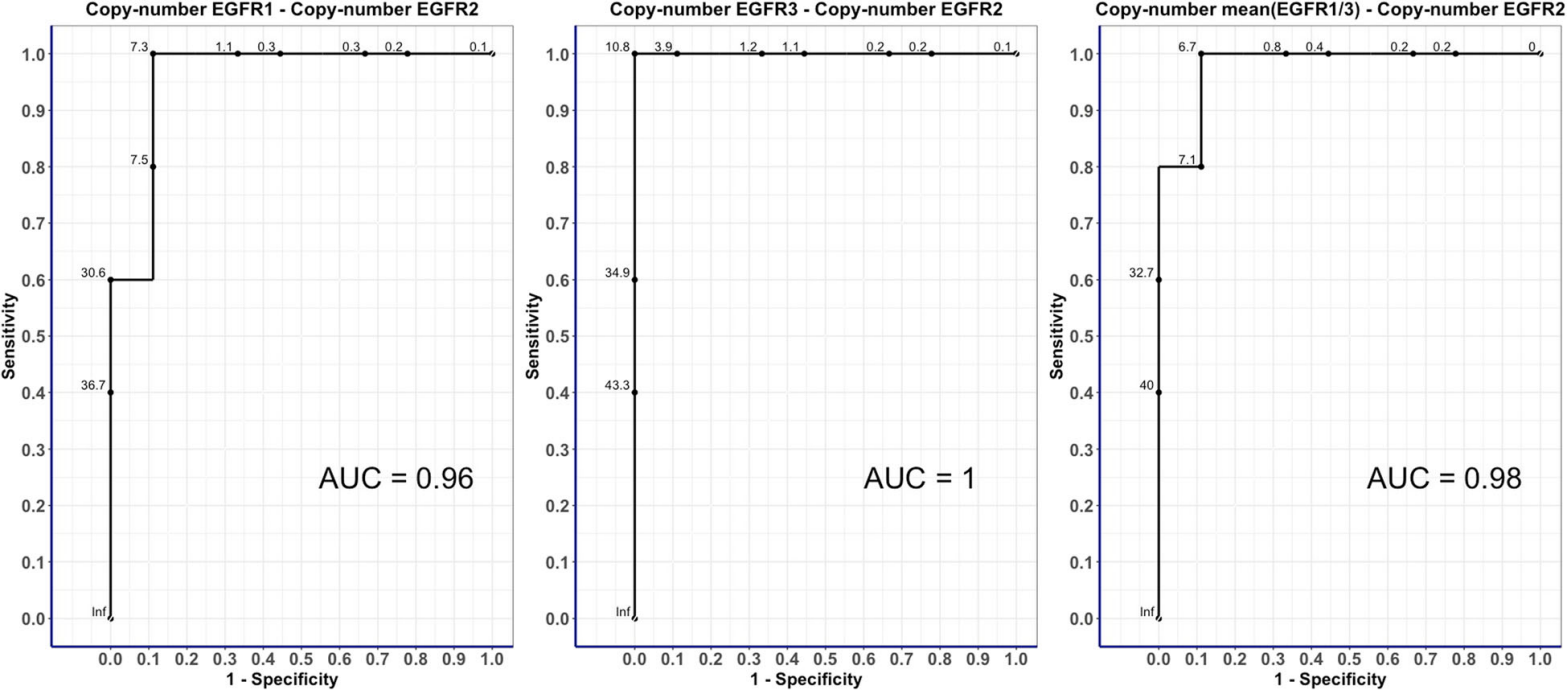
ROC curves of the copy number differences between the three dPCR assays for the prediction of the *EGFRvIII* variant. The three ROC curves represent the identification of the best diagnostic test to identify *EGFRvIII* using the absolute copy number differences between EGFR2 and the other dPCR assays, namely, EGFR1, EGFR3 and mean (EGFR1 + EGFR3). The best predictive test was selected using the highest AUC (difference of EGFR3 and EGFR2) and the threshold of the copy number difference that maximizes the sensitivity and specificity (10.8)

A total of 19 patients were tested for the *EGFRvIII* variant. Among them, five patients were positive for an *EGFRvIII* variant using LD-RT-PCR. The dPCR assays detected a total of 5/19 patients with the *EGFRvIII* variant, all of which were identical to those detected by the LD-RT-PCR method (Fig. 2a and Additional File: Fig. S2). We observed that the most predictive copy number differences between dPCR assays for detecting the *EGFRvIII* variant was between the EGFR3 and EGFR2 assays, rather than between the EGFR1 and EGFR2 assays, with a copy number absolute difference of 10.8 (AUC 1) (Fig. 3).

**Fig. 4:**
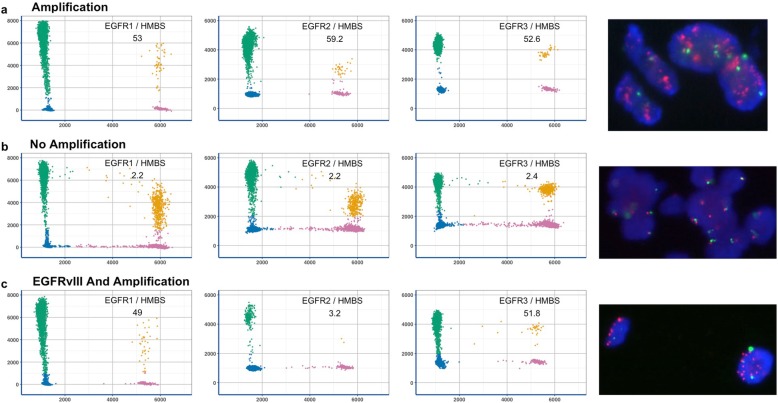
Three illustrative sample tumor examples using three dPCR assays and FISH. **a** Tumor with *EGFR* amplification. **b** Tumor without *EGFR* amplification. **c** Tumor with *EGFR* amplification and concomitant *EGFRvIII* variant. Corresponding FISH images are shown at the end of the line on the right

Taken together, using these results, the EGFR3 assay and the EGFR2/EGFR3 assay were selected to detect *EGFR*-amplified glioblastoma and to identify the *EGFRvIII* variant in the validation cohort (Fig. 4).

### Results of the dPCR assays in the validation cohort

A total of 43 patients were included in the validation cohort. Among them, 19/43 (44%) were *EGFR*-amplified *IDH-*wt glioblastomas using FISH (19/43, 44%). Using the dPCR assay, based on the EGFR3 assay, the same 19 patients with an *EGFR* amplification were identified, leading to a sensitivity, specificity, positive and negative predictive values of 100%. The mean estimated copy number was 56.9 (range 13.6–196.5), and the median was 48.7. The EGFR3 assay allowed for the identification of *EGFR* copy gain in 16 *EGFR*-non-amplified glioblastomas with a mean copy number of 3.3 (range 2.6–3.9). A single patient harbored an *EGFR* amplification in 5% of tumor cell nuclei (Additional File: Fig. S3), and this was considered non-amplified both by FISH and by dPCR. Among the 19 *EGFR*-amplified glioblastomas, *EGFRvIII* was identified in 8 patients by dPCR EGFR2/EGFR3 assay, and all were confirmed using LD-RT-PCR.

Interestingly, two *EGFR*-amplified glioblastomas, identified by dPCR and confirmed by FISH, had very low amount of DNA (2 ng and 6 ng). One tumor had concomitant *EGFRvIII* variant confirmed by LD-RT-PCR, highlighting the value of the dPCR-based method for glioblastoma samples with small amount of DNA.

### Cost estimation of dPCR

The estimated cost for one patient (CNV detected in EGFR1, EGFR2 and EGFR3) was 43% lower using dPCR than FISH (60.88€: 30.33€ for reagents and 30.55€ for working time) for dPCR and 106.01€ for FISH) (Additional File: Table S4). The total cost of dPCR decreased to 50.77€ when only EGFR2 and EGFR3 assays were used. Moreover, dPCR assays with EGFR2/EGFR3 detect both the *EGFR* amplification and *EGFRvIII* variant, whereas the FISH assay can only identify an *EGFR* amplification.

## Discussion

This study shows that the specific dPCR assay using LNA-hydrolysis probes from UPL® is a reliable and simple method to simultaneously detect an *EGFR* amplification and *EGFRvIII* variant, and this can be used in clinical practice in glioblastoma. Indeed, using an experimental design based on two independent cohorts, we showed that the dPCR assay was better than standard methods and was able to detect the main somatic *EGFR* alterations in DNA extracted from FFPE tumor samples with a diagnostic performance of 100%.

The current molecular findings in our work were similar to those previously published using larger cohorts of glioblastoma patients eligible for Stupp treatment [22, 23]. Indeed, the overall proportion of *EGFR*-amplified tumors is similar to that reported in the TCGA (43%) [4], especially when accounting for the criteria recently suggested by French et al. to classify tumors as *EGFR* amplified (*EGFR* copy number higher than 5 and more than 50% of the nuclei were amplified) [7]. Moreover, it has also been recently confirmed that the proportion of patients with *EGFR-*amplified glioblastoma using NGS, FFPE-based or CGH-array techniques is between 35 and 45% [24, 25]. One of the major strengths of our study is that, in addition to the qualitative assessment of *EGFR* amplification, the dPCR assay using LNA-hydrolysis probes also provides a reliable quantitative copy number estimation compared to NGS. We also confirmed the high number of *EGFR* copy number amplicons in glioblastoma, including 13% of tumors (*n* = 8) with greater than 50 copy gains. Although the therapeutic impact of high *EGFR*-amplified tumors remains to be evaluated, our results clearly showed that our dPCR assay may be used to screen the *EGFR* copy number for decision making, particularly in further studies focusing on therapies targeting this molecular alteration.

One of the other benefits of the dPCR assay developed in our work is its potential economic cost compared to FISH. For one patient, the cost decreases from 40 to 50% when using only the EGFR2/EGFR3 assay. Moreover, in contrast to FISH, dPCR allows the simultaneous detection of the *EGFRvIII* variant, which has been shown to be a potential therapeutic target [26]. Lower copy number values observed between EGFR2 and EGFR3 amplicons are very likely explained by the presence of the *EGFRvIII* variant. The EGFR3 amplicon is not located in a specific gene region affected by recurrent splicing variants or deletions but is located between exon 25 and the C-terminal region. The qualitative discrepancy between EGFR1 and EGFR2 amplicons for detecting the gene copy number from the same tumor is probably due to breakpoint variability of the *EGFRvIII* variant. The EGFR1 amplicon is located at the start of intron 1 in an area containing various breakpoints for the *EGFRvIII* splicing variant [27]. The EGFR1 amplicon may therefore match with tumor DNA if the breakpoint is closer to exon 2 but may mismatch with the tumor DNA if the breakpoint is closer to exon 1. As shown in our results, the value of using EGFR2 resides in its location within the spliced area, regardless of the breakpoint. Therefore, the comparison of the copy number estimation using EGFR2 and EGFR3 assays should be a more sensitive method than dPCR using an amplicon located at the end of exon 1 [27, 28].

NGS-based CNV identification using panels dedicated to glioblastoma has been demonstrated to be as sensitive as FISH or CGH array [25, 29]. In our exploratory cohort, the diagnostic performance for the detection of *EGFR* amplification was 100% when comparing dPCR and *EGFR*-targeted NGS. The major advantage of NGS resides in the fact that a single assay may detect somatic point mutations and multiple CNVs. However, the cost of a single NGS assay remains high, which currently hampers its routine use. Moreover, it has also been reported that multiple CNVs may be easily detected with the LNA-probe hydrolysis dPCR method without any proportional cost increase, for example, the concomitant detection of other amplicons located on *MET, PDGFRA, KIT, AKT1* or *CDKN2A* homozygous deletion [30]. Moreover, the quantity of tumor DNA necessary is lower for dPCR than for NGS, making this technique more suitable for small tumor fragments, including those derived from biopsies or fragments containing low amounts of tumor DNA, notably in the case of tumor necrosis. Detection of CNV by NGS requires the comparison between patient-matched and unmatched normal tissue. In the specific situation of *EGFR* amplification detection in glioblastoma sample, the ideal comparison tissue should have been healthy brain tissue, which is virtually impossible to obtain in daily practice [31, 32]. The major advantage of dPCR use is that there is no need for healthy brain tissue since *HMBS* reference gene is not altered in tumor samples.

At last, our results are based on experiments using LNA-probes provided by Roche®. The experimental procedure is not restricted to specific manufacturer’s probes and could easily be used with other manufacturers’ LNA-probes provided that these probes are designed to be used at a hybridization temperature of 56 °C.

In conclusion, our results highlight that the dPCR assay using LNA-hydrolysis probes allowed the simultaneous detection of the *EGFR* amplification and *EGFRvIII* variant and may be used routinely in patients treated for glioblastoma.

## Supplementary information

**Additional file 1: Supplementary Table**. Details of *EGFR* amplicons by NGS.

**Additional file 2: Supplementary Data**. Results of LD-RT-PCR for *EGFRvIII* detection.

**Additional file 3.**

**Additional file 4: Supplementary Material**: cost evaluation of FISH method.

## Data Availability

Deidentified data are available on request.

## Abbreviations

aCGH: Array-comparative genomic hybridization
CNV: Copy number variation
dPCR: Digital PCR
ddPCR: Droplet digital PCR
*EGFR*: *Epidermal Growth Factor Receptor*
*EGFRvIII*: *EGFR* variant III
FISH: Fluorescence in situ hybridization
*HMBS*: *Hydroxymethylbilane synthase*
LD-RT-PCR: Ligation-dependent reverse transcription polymerase chain reaction
LNA: Locked nucleic acid
*IDH*: *Isocitrate dehydrogenase*
*MGMT*: *O-6-methylguanine-DNA methyltransferase*
NGS: Next-generation sequencing
ROC: Recursive operating curve
*TERT*: *Telomerase reverse transcriptase*
UPL: Universal Probe Library®
wt: Wild-type
