# Supplementary material for: Simultaneous detection of EGFR amplification and EGFRvIII variant using digital PCR-based method in glioblastoma
Source: Acta Neuropathol Commun. 2020 Apr 17;8:52. doi: 10.1186/s40478-020-00917-6 (PMC7165387; doi:10.1186/s40478-020-00917-6)
Supplement: Supplementary file 1 — Additional file 1: Supplementary Table. Details of EGFR amplicons by NGS. [file 40478_2020_917_MOESM1_ESM.docx]

**Supplementary Table. Details of *EGFR* amplicons by NGS**

| Chromosome | Start position, reference=hg19 | End position, reference=hg19 | Reference |
| --- | --- | --- | --- |
| chr7 | 55221705 | 55221800 | AMPL7154411437 |
| chr7 | 55221820 | 55221914 | AMPL7154534279 |
| chr7 | 55220212 | 55220307 | AMPL7154631416 |
| chr7 | 55229199 | 55229299 | AMPL7154631417 |
| chr7 | 55214301 | 55214380 | AMPL7154631422 |
| chr7 | 55223510 | 55223605 | AMPL7154631975 |
| chr7 | 55227869 | 55227965 | AMPL7154632027 |
| chr7 | 55270238 | 55270334 | AMPL7154632162 |
| chr7 | 55232961 | 55233053 | AMPL7154644020 |
| chr7 | 55231450 | 55231544 | AMPL7154800977 |
| chr7 | 55210028 | 55210118 | AMPL7156070426 |
| chr7 | 55269425 | 55269511 | AMPL7156576042 |
| chr7 | 55211040 | 55211128 | AMPL7156584305 |
| chr7 | 55086948 | 55087045 | AMPL7158525723 |
| chr7 | 55218947 | 55219042 | AMPL7159478226 |
| chr7 | 55240763 | 55240882 | AMPLP227722022 |
