# Supplementary material for: Simultaneous detection of EGFR amplification and EGFRvIII variant using digital PCR-based method in glioblastoma
Source: Acta Neuropathol Commun. 2020 Apr 17;8:52. doi: 10.1186/s40478-020-00917-6 (PMC7165387; doi:10.1186/s40478-020-00917-6)
Supplement: Supplementary file 2 — Additional file 2: Supplementary Data. Results of LD-RT-PCR for EGFRvIII detection. [file 40478_2020_917_MOESM2_ESM.docx]

**Supplementary Data. Results of LD-RT-PCR for *EGFRvIII* detection.**

Plots are showing three columns as horizontal axis for exon 1 – exon 2 ligation (EGFR1_2), exon 1 – exon 8 (EGFR1_8) and exon 7 – exon 8 (EGFR7_8). In case of *EGFRvIII* variant ligations are represented in the three columns.

| Patient #02 | Patient #04 | Patient #07 |
| --- | --- | --- |
|  |  |  |

| Patient #08 | Patient #10 | Patient #60 |
| --- | --- | --- |
|  |  |  |
| Patient #62 | Patient #63 | Patient #70 |
|  |  | **** |
