# Supplementary figures and images for: Simultaneous detection of EGFR amplification and EGFRvIII variant using digital PCR-based method in glioblastoma
Source: Acta Neuropathol Commun. 2020 Apr 17;8:52. doi: 10.1186/s40478-020-00917-6 (PMC7165387; doi:10.1186/s40478-020-00917-6)

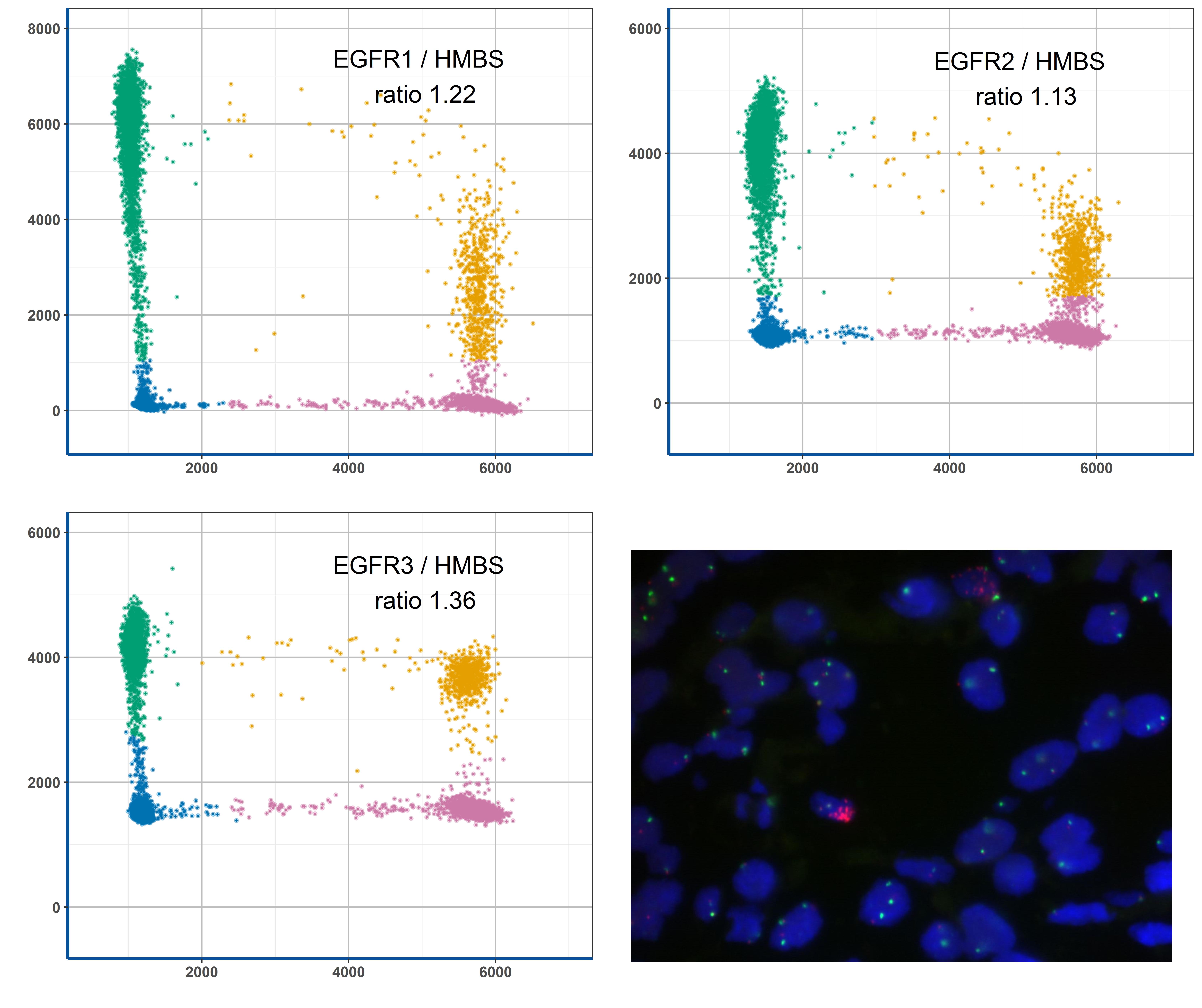

Supplement: Supplementary file 3 — Additional file 3. [file 40478_2020_917_MOESM3_ESM.jpeg]
