# Supplementary material for: Simultaneous detection of EGFR amplification and EGFRvIII variant using digital PCR-based method in glioblastoma
Source: Acta Neuropathol Commun. 2020 Apr 17;8:52. doi: 10.1186/s40478-020-00917-6 (PMC7165387; doi:10.1186/s40478-020-00917-6)
Supplement: Supplementary file 4 — Additional file 4: Supplementary Material: cost evaluation of FISH method. [file 40478_2020_917_MOESM4_ESM.docx]

**Supplementary Material: cost evaluation of FISH method**

Cost is estimated for FISH method based on current practice, manufacturer instructions and public prices of the providers.

**Reagent cost**

| **Provider** | **Product** | **Reference** | **Price (**€) | **Vol / nb. of test** | **Number of total tests** | **For one patient**  **(€)** |
| --- | --- | --- | --- | --- | --- | --- |
| ABBOTT | Vysis paraffin pretreatment IV | 01N31-005 | 431.86 | 5x12 slides | 62 | 7.20 |
| ABBOTT | Vysis EGFR/CEP7 FISH probe kit | 01N35-020 | 1647.13 | 20 slides | 62 | 82.35 |
| ABBOTT | DAPI I | 06J49-001 | 88.33 | 10µl/slide | 62 | 0.88 |
| Total | | | | | | 90.43 |

**Working time**

Cost are calculated based on French charged salaries: technician (cost per hour: 21.67€) and clinical biologist (cost per hour: 47.48€)

| **Step** | **Human Ressource** | **Time (h)** | **Price** (€) |
| --- | --- | --- | --- |
| Preparation of Experience plan | technician | 0.2 | 4.33 |
| FISH protocol | technician | 0.2 | 4.33 |
| Technical verification | technician | 0.1 | 2.17 |
| Interpretation | clinical biologist | 0.1 | 4.75 |
| Total | | | 15.58 |
